# 4,4-Di­fluoro-2,3;5,6-bis­(tetra­methylene)-4-bora-3a,4a-di­aza-*s*-indacene (LD540)

**DOI:** 10.1107/S1600536813033448

**Published:** 2013-12-18

**Authors:** Kirsi Salorinne, Tiia-Riikka Tero, Tanja Lahtinen

**Affiliations:** a Department of Chemistry, Nanoscience Center, University of Jyväskylä, PO Box 35, FIN-40014 University of Jyväskylä, Finland

## Abstract

The title compound, C_18_H_21_BF_2_N_2_, is a lipophilic dye based on a BODIPY fluoro­phore backbone, which was developed for microscopic imaging of lipid droplets; the mol­ecule has a planar BODIPY core [dihedral angle between the pyrrole rings = 2.3 (3)°] and two tetra­methyl­ene substituents at the 2,3- and 5,6-positions in a half-chair conformation. One of the tetra­methyl­ene substituents is disordered over two two sets of sites with site occupancies of 0.5. In the crystal, pairs of C—H⋯F inter­actions link the mol­ecules into inversion dimers. Neighbouring dimers are linked by further C—H⋯F inter­actions, forming an infinite array. C—H⋯π and π–π [centroid–centroid distance = 4.360 (3) Å] inter­actions are observed between the BODIPY core and the tetra­methyl­ene substituents of neighbouring dimer pairs.

## Related literature   

For lipid droplets and fluorescence imaging with LD540, see: Beller *et al.* (2010 ▶); Bickel *et al.* (2009 ▶); Spandl *et al.* (2009 ▶). For related BODIPY structures, see: Uppal *et al.* (2012 ▶).
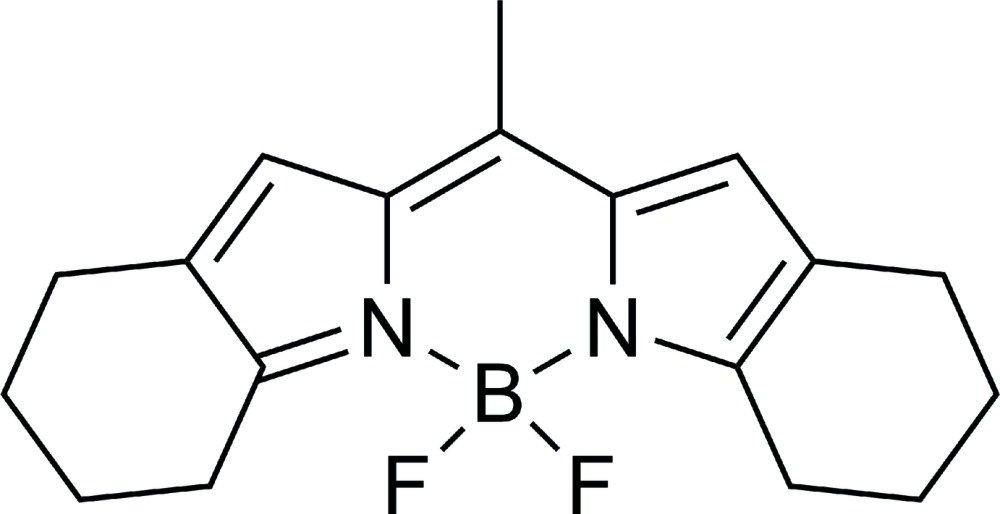


## Experimental   

### 

#### Crystal data  

C_18_H_21_BF_2_N_2_
*M* *_r_* = 314.18
Monoclinic, 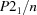

*a* = 8.8836 (4) Å
*b* = 16.467 (1) Å
*c* = 11.4865 (6) Å
β = 111.271 (3)°
*V* = 1565.84 (15) Å^3^
*Z* = 4
Cu *K*α radiation
μ = 0.77 mm^−1^
*T* = 173 K
0.1 × 0.1 × 0.04 mm

#### Data collection  

Nonius KappaCCD diffractometer with APEXII detector
Absorption correction: multi-scan (*SADABS*; Bruker, 2004 ▶) *T*_min_ = 0.840, *T*_max_ = 1
7413 measured reflections
2511 independent reflections
1902 reflections with *I* > 2σ(*I*)
*R*_int_ = 0.054

#### Refinement  

*R*[*F* ^2^ > 2σ(*F* ^2^)] = 0.047
*wR*(*F* ^2^) = 0.125
*S* = 1.03
2511 reflections
227 parameters
H-atom parameters constrained
Δρ_max_ = 0.17 e Å^−3^
Δρ_min_ = −0.30 e Å^−3^

### 

Data collection: *COLLECT* (Bruker, 2004 ▶); cell refinement: *SCALEPACK* (Otwinowski & Minor, 1997 ▶); data reduction: *DENZO* (Otwinowski & Minor, 1997 ▶) and *SCALEPACK*; program(s) used to solve structure: *SHELXS97* (Sheldrick, 2008 ▶); program(s) used to refine structure: *SHELXL97* (Sheldrick, 2008 ▶); molecular graphics: *OLEX2* (Dolomanov *et al.*, 2009 ▶); software used to prepare material for publication: *OLEX2*.

## Supplementary Material

Crystal structure: contains datablock(s) global, I. DOI: 10.1107/S1600536813033448/vm2201sup1.cif

Structure factors: contains datablock(s) I. DOI: 10.1107/S1600536813033448/vm2201Isup2.hkl

Supporting information file. DOI: 10.1107/S1600536813033448/vm2201Isup3.mol

Additional supporting information:  crystallographic information; 3D view; checkCIF report

## Figures and Tables

**Table 1:** Hydrogen-bond geometry (Å, °) *Cg*1 and *Cg*2 are the centroids of the N4,C5,C10–C12 and N22,C21,C14–C16 rings, respectively.

*D*—H⋯*A*	*D*—H	H⋯*A*	*D*⋯*A*	*D*—H⋯*A*
C23—H23*B*⋯F3^i^	0.96	2.66	3.621 (3)	178
C8—H8*B*⋯F2^ii^	0.97	2.56	3.252 (3)	129
C17—H17*A*⋯*Cg*2^iii^	0.97	3.10	3.879 (3)	138

Symmetry codes: (i) 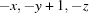; (ii) 
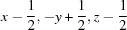
; (iii) 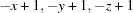.

**Fig. 1.**
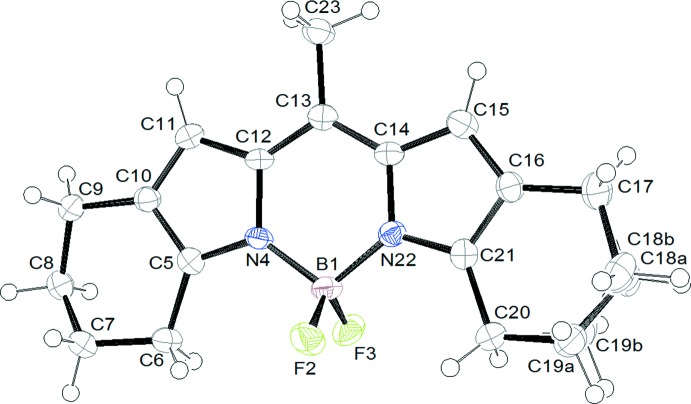
Molecular structure of the title compound with atom labels and 50% probability displacement ellipsoids for non-H atoms.

**Fig. 2.**
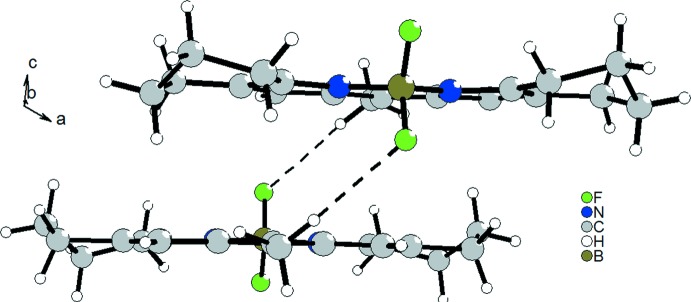
Dimer pair formed by the intermolecular F···H—C interactions (black dotted line) between the opposite facing molecules.

**Fig. 3.**
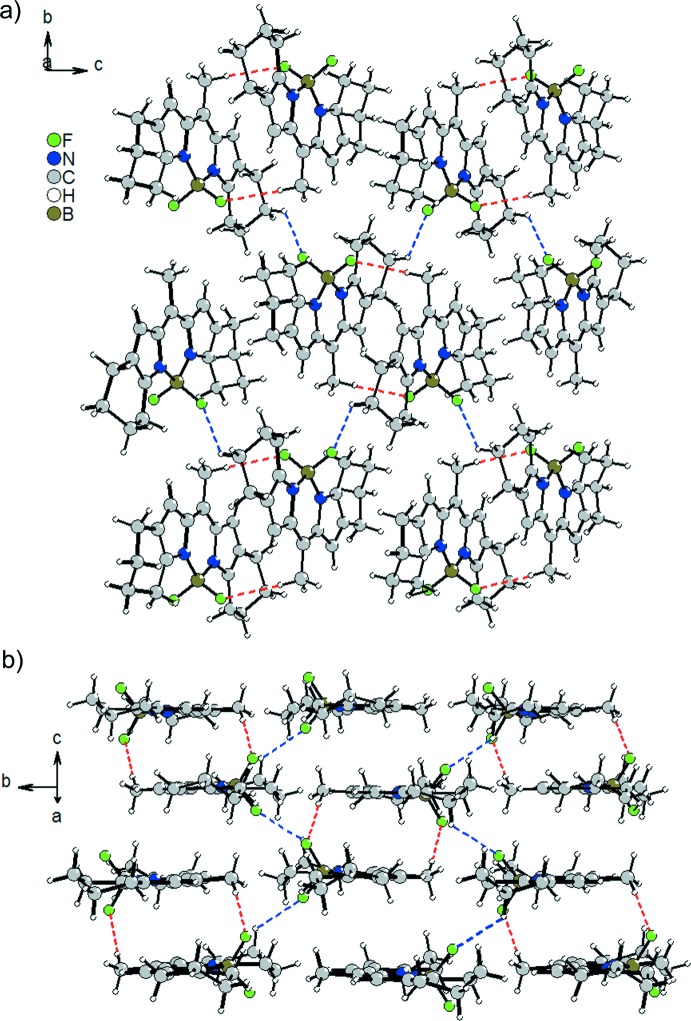
Packing diagram showing the infinite array of dimer pairs in the crystal lattice connected by the intermolecular F···H—C interactions viewed along a) the a axis and b) from a view highlighting the aromatic interactions formed between the molecule layers. Intermolecular F···H—C interactions forming the dimer pairs have been marked with red and the ones between the dimer pairs have been marked with blue color.

## supplementary crystallographic information

### 1. Comment

Lipid droplets are metabolically active organelles (Beller *et al.*, 2010; Bickel *et al.*, 2009), which function as intracellular storehouses of lipid esters found inside almost all cells. LD540 is one of the dyes that can be used for multicolor fluorescence imaging for lipid droplets in both fixed and living cells (Spandl *et al.*, 2009). In the structure of the title compound, the BODIPY core is planar having the average dihedral angle formed between the two pyrrole rings of 2.3 (3)° (Fig. 1). The two tetramethylene substituents on either side of the BODIPY core at the 2,3- and 5,6-positions are in a half-chair conformation. Intermolecular F···H—C interactions (distance of 2.661 (3) Å) between the fluoride (F3) and methyl (C23) groups of the opposite facing molecules connect the two LD540 molecules to form a dimer (Fig.2, Table 2). In a similar manner, the second fluoride (F2) atom forms an intermolecular F···H—C interaction (distance of 2.555 (3) Å) to one of the CH_2_ (C8) groups of the tetramethylene unit connecting the neighbouring dimer pairs in an infinite array through the crystal lattice (Fig. 3, Table 2). In addition to the F···H—C interactions, intermolecular C—H···π interactions [C18A—H18A···*Cg1*^i^ = 2.812 Å and C17—H17A···*Cg2*^ii^ = 3.103 Å; *Cg1* and *Cg2* are the centroids of rings N4,C5,C10-C12 and N22,C21,C14-C16, respectively; symmetry codes: (i) *x* + 1, *y*, *z*; (ii) -*x*, -*y* + 1, -*z* + 1] and π···π interactions [*Cg2*···*Cg2*^iii^ = 4.360 (3) Å; symmetry code: (iii) -*x* + 1, -*y* + 1, -*z* +1] are observed between the BODIPY core and the tetramethylene substituents of the neighbouring dimer pairs.

### 2. Experimental

The title compound was synthesized by a known method described by Christoph Thiele and co-workers (Spandl *et al.*, 2009) using tetrahydropyrrole, acetylchloride and BF_3_-etherate as the starting material. For single-crystal X-ray analysis the crude product was recrystallized from dichloromethane yielding greenish red prism crystals.

### 3. Refinement

All H atoms were visible in the electron density maps, but those bonded to C were ideally positioned and allowed to ride on their parent atoms with *U*_iso_(H) of 1.2 (or 1.5 for methyl) times *U*_eq_(C). One of the tetramethylene substituent is disordered over two positions (C18—C19) having fixed site occupation factors of 0.5.

### Crystal data

**Table:**

C_18_H_21_BF_2_N_2_	*F*(000) = 664
*M**_r_* = 314.18	*D*_x_ = 1.333 Mg m^−^^3^
Monoclinic, *P*2_1_/*n*	Cu *K*α radiation, λ = 1.54184 Å
*a* = 8.8836 (4) Å	Cell parameters from 2349 reflections
*b* = 16.467 (1) Å	θ = 0.9–62.4°
*c* = 11.4865 (6) Å	µ = 0.77 mm^−^^1^
β = 111.271 (3)°	*T* = 173 K
*V* = 1565.84 (15) Å^3^	Prism, green red
*Z* = 4	0.1 × 0.1 × 0.04 mm

### Data collection

**Table:**

Nonius KappaCCD diffractometer with APEXII detector	2511 independent reflections
Radiation source: Enraf–Nonius FR590	1902 reflections with *I* > 2σ(*I*)
Horizonally mounted graphite crystal monochromator	*R*_int_ = 0.054
Detector resolution: 9 pixels mm^-1^	θ_max_ = 63.3°, θ_min_ = 4.9°
CCD rotation images, thick slices scans	*h* = −10→10
Absorption correction: multi-scan (*SADABS*; Bruker, 2004)	*k* = −19→15
*T*_min_ = 0.840, *T*_max_ = 1	*l* = −11→13
7413 measured reflections	

### Refinement

**Table:**

Refinement on *F*^2^	Primary atom site location: structure-invariant direct methods
Least-squares matrix: full	Secondary atom site location: difference Fourier map
*R*[*F*^2^ > 2σ(*F*^2^)] = 0.047	Hydrogen site location: inferred from neighbouring sites
*wR*(*F*^2^) = 0.125	H-atom parameters constrained
*S* = 1.03	*w* = 1/[σ^2^(*F*_o_^2^) + (0.0626*P*)^2^ + 0.2776*P*] where *P* = (*F*_o_^2^ + 2*F*_c_^2^)/3
2511 reflections	(Δ/σ)_max_ < 0.001
227 parameters	Δρ_max_ = 0.17 e Å^−^^3^
0 restraints	Δρ_min_ = −0.30 e Å^−^^3^

### Special details

**Table:**

Experimental. SADABS v.2.03 (Bruker, 2004) was used for absorption correction. R(int) was 0.0552 before and 0.0509 after correction. The Ratio of minimum to maximum transmission is 0.8396. The λ/2 correction factor is 0.0015.
Geometry. All e.s.d.'s (except the e.s.d. in the dihedral angle between two l.s. planes) are estimated using the full covariance matrix. The cell e.s.d.'s are taken into account individually in the estimation of e.s.d.'s in distances, angles and torsion angles; correlations between e.s.d.'s in cell parameters are only used when they are defined by crystal symmetry. An approximate (isotropic) treatment of cell e.s.d.'s is used for estimating e.s.d.'s involving l.s. planes.

### Fractional atomic coordinates and isotropic or equivalent isotropic displacement parameters (Å^2^)

**Table:**

	*x*	*y*	*z*	*U*_iso_*/*U*_eq_	Occ. (<1)
C5	−0.1259 (3)	0.36857 (12)	0.10617 (19)	0.0289 (5)	
C6	−0.1499 (3)	0.27857 (12)	0.0961 (2)	0.0327 (5)	
H6A	−0.0857	0.2531	0.1746	0.039*	
H6B	−0.1142	0.2575	0.0315	0.039*	
C7	−0.3294 (3)	0.25826 (13)	0.0640 (2)	0.0388 (6)	
H7A	−0.3488	0.2023	0.0361	0.047*	
H7B	−0.3557	0.2636	0.1386	0.047*	
C8	−0.4386 (3)	0.31373 (13)	−0.0374 (2)	0.0395 (6)	
H8A	−0.5498	0.2961	−0.0598	0.047*	
H8B	−0.4103	0.3094	−0.1111	0.047*	
C9	−0.4245 (3)	0.40230 (13)	0.0048 (2)	0.0347 (5)	
H9A	−0.4776	0.4370	−0.0667	0.042*	
H9B	−0.4780	0.4095	0.0642	0.042*	
C10	−0.2501 (2)	0.42636 (12)	0.06426 (19)	0.0286 (5)	
C11	−0.1770 (3)	0.50178 (12)	0.09029 (19)	0.0296 (5)	
H11	−0.2295	0.5517	0.0724	0.036*	
C12	−0.0099 (3)	0.48998 (12)	0.14838 (19)	0.0282 (5)	
C13	0.1168 (2)	0.54569 (12)	0.19475 (18)	0.0287 (5)	
C14	0.2753 (3)	0.51881 (12)	0.24992 (19)	0.0291 (5)	
C15	0.4217 (3)	0.56116 (13)	0.30543 (19)	0.0321 (5)	
H15	0.4335	0.6173	0.3117	0.039*	
C16	0.5448 (3)	0.50481 (13)	0.3490 (2)	0.0320 (5)	
C17	0.7244 (3)	0.51515 (14)	0.4149 (2)	0.0395 (6)	
H17A	0.7478	0.5309	0.5011	0.047*	0.5
H17B	0.7635	0.5576	0.3745	0.047*	0.5
H17C	0.7707	0.5363	0.3564	0.047*	0.5
H17D	0.7446	0.5543	0.4819	0.047*	0.5
C18A	0.8118 (8)	0.4330 (5)	0.4104 (6)	0.0365 (15)	0.5
H18A	0.8079	0.4234	0.3260	0.044*	0.5
H18B	0.9244	0.4365	0.4650	0.044*	0.5
C19A	0.7297 (6)	0.3617 (3)	0.4520 (5)	0.0322 (12)	0.5
H19A	0.7932	0.3126	0.4606	0.039*	0.5
H19B	0.7219	0.3738	0.5323	0.039*	0.5
C18B	0.8044 (9)	0.4370 (5)	0.4674 (6)	0.0435 (17)	0.5
H18C	0.9193	0.4420	0.4848	0.052*	0.5
H18D	0.7905	0.4269	0.5460	0.052*	0.5
C19B	0.7418 (7)	0.3665 (4)	0.3843 (6)	0.0488 (14)	0.5
H19C	0.8031	0.3187	0.4236	0.059*	0.5
H19D	0.7597	0.3757	0.3069	0.059*	0.5
C20	0.5599 (3)	0.34915 (14)	0.3530 (2)	0.0362 (5)	
H20A	0.5688	0.3252	0.2786	0.043*	0.5
H20B	0.4993	0.3120	0.3849	0.043*	0.5
H20C	0.5202	0.3118	0.2832	0.043*	0.5
H20D	0.5433	0.3250	0.4244	0.043*	0.5
C21	0.4724 (2)	0.42804 (13)	0.32034 (19)	0.0292 (5)	
C23	0.0777 (3)	0.63483 (12)	0.1889 (2)	0.0341 (5)	
H23A	0.0355	0.6480	0.2526	0.051*	
H23B	−0.0014	0.6476	0.1084	0.051*	
H23C	0.1740	0.6658	0.2019	0.051*	
B1	0.1873 (3)	0.36599 (14)	0.2105 (2)	0.0315 (6)	
N4	0.0190 (2)	0.40608 (10)	0.15627 (15)	0.0280 (4)	
N22	0.3114 (2)	0.43595 (10)	0.26012 (15)	0.0288 (4)	
F2	0.19599 (15)	0.31382 (7)	0.30739 (12)	0.0441 (4)	
F3	0.21955 (15)	0.32285 (7)	0.11765 (12)	0.0430 (4)	

### Atomic displacement parameters (Å^2^)

**Table:**

	*U*^11^	*U*^22^	*U*^33^	*U*^12^	*U*^13^	*U*^23^
C5	0.0323 (12)	0.0247 (11)	0.0298 (11)	−0.0005 (9)	0.0115 (9)	0.0000 (8)
C6	0.0360 (12)	0.0236 (11)	0.0368 (12)	−0.0002 (9)	0.0111 (10)	−0.0019 (9)
C7	0.0404 (13)	0.0267 (12)	0.0519 (15)	−0.0042 (10)	0.0200 (11)	−0.0017 (10)
C8	0.0315 (12)	0.0306 (12)	0.0544 (15)	−0.0031 (10)	0.0134 (11)	−0.0061 (11)
C9	0.0303 (12)	0.0314 (12)	0.0428 (13)	0.0004 (9)	0.0135 (10)	−0.0012 (10)
C10	0.0288 (11)	0.0260 (11)	0.0313 (11)	0.0024 (9)	0.0112 (9)	−0.0001 (9)
C11	0.0314 (12)	0.0233 (10)	0.0330 (11)	0.0042 (9)	0.0103 (10)	0.0014 (9)
C12	0.0324 (12)	0.0224 (10)	0.0292 (11)	0.0022 (9)	0.0104 (9)	0.0012 (9)
C13	0.0339 (12)	0.0237 (11)	0.0281 (11)	0.0001 (9)	0.0107 (9)	0.0002 (9)
C14	0.0318 (11)	0.0243 (11)	0.0297 (11)	0.0003 (9)	0.0093 (9)	0.0012 (9)
C15	0.0340 (12)	0.0257 (11)	0.0345 (12)	−0.0033 (9)	0.0096 (10)	0.0000 (9)
C16	0.0300 (12)	0.0331 (12)	0.0319 (12)	−0.0005 (9)	0.0099 (10)	−0.0005 (9)
C17	0.0324 (12)	0.0399 (14)	0.0435 (14)	−0.0035 (10)	0.0105 (11)	−0.0020 (11)
C18A	0.027 (3)	0.043 (3)	0.040 (4)	−0.001 (2)	0.013 (3)	−0.010 (4)
C19A	0.030 (3)	0.034 (3)	0.031 (3)	0.009 (2)	0.009 (2)	0.003 (2)
C18B	0.034 (3)	0.047 (4)	0.044 (4)	0.003 (2)	0.008 (3)	−0.002 (4)
C19B	0.032 (3)	0.043 (3)	0.063 (4)	0.003 (2)	0.008 (3)	−0.003 (3)
C20	0.0324 (12)	0.0321 (12)	0.0413 (13)	0.0045 (10)	0.0100 (10)	0.0010 (10)
C21	0.0289 (11)	0.0308 (12)	0.0273 (11)	0.0026 (9)	0.0095 (9)	0.0007 (9)
C23	0.0353 (12)	0.0242 (12)	0.0384 (12)	0.0002 (9)	0.0081 (10)	0.0009 (9)
B1	0.0327 (14)	0.0216 (12)	0.0364 (13)	0.0028 (10)	0.0081 (11)	0.0007 (11)
N4	0.0303 (10)	0.0214 (9)	0.0309 (9)	0.0006 (7)	0.0094 (8)	0.0004 (7)
N22	0.0303 (10)	0.0243 (9)	0.0305 (9)	0.0025 (7)	0.0096 (8)	0.0005 (7)
F2	0.0377 (8)	0.0341 (7)	0.0515 (8)	−0.0011 (6)	0.0055 (6)	0.0174 (6)
F3	0.0339 (7)	0.0379 (7)	0.0521 (8)	0.0047 (5)	0.0094 (6)	−0.0171 (6)

### Geometric parameters (Å, º)

**Table:**

C5—C6	1.496 (3)	C17—H17C	0.9700
C5—C10	1.403 (3)	C17—H17D	0.9700
C5—N4	1.353 (3)	C17—C18A	1.570 (7)
C6—H6A	0.9700	C17—C18B	1.488 (8)
C6—H6B	0.9700	C18A—H18A	0.9700
C6—C7	1.538 (3)	C18A—H18B	0.9700
C7—H7A	0.9700	C18A—C19A	1.548 (10)
C7—H7B	0.9700	C19A—H19A	0.9700
C7—C8	1.521 (3)	C19A—H19B	0.9700
C8—H8A	0.9700	C19A—C20	1.539 (6)
C8—H8B	0.9700	C18B—H18C	0.9700
C8—C9	1.527 (3)	C18B—H18D	0.9700
C9—H9A	0.9700	C18B—C19B	1.477 (10)
C9—H9B	0.9700	C19B—H19C	0.9700
C9—C10	1.502 (3)	C19B—H19D	0.9700
C10—C11	1.383 (3)	C19B—C20	1.549 (6)
C11—H11	0.9300	C20—H20A	0.9700
C11—C12	1.403 (3)	C20—H20B	0.9700
C12—C13	1.399 (3)	C20—H20C	0.9700
C12—N4	1.402 (3)	C20—H20D	0.9700
C13—C14	1.390 (3)	C20—C21	1.490 (3)
C13—C23	1.504 (3)	C21—N22	1.350 (3)
C14—C15	1.408 (3)	C23—H23A	0.9600
C14—N22	1.397 (3)	C23—H23B	0.9600
C15—H15	0.9300	C23—H23C	0.9600
C15—C16	1.382 (3)	B1—N4	1.544 (3)
C16—C17	1.507 (3)	B1—N22	1.553 (3)
C16—C21	1.402 (3)	B1—F2	1.385 (3)
C17—H17A	0.9700	B1—F3	1.395 (3)
C17—H17B	0.9700		
			
H18A···Cg(1)^i^	2.812	Cg(2)···Cg(2)^iii^	4.360 (3)
H17A···Cg(2)^ii^	3.103		
			
C10—C5—C6	124.97 (19)	C18B—C17—H17D	109.3
N4—C5—C6	124.87 (19)	C17—C18A—H18A	109.6
N4—C5—C10	110.15 (18)	C17—C18A—H18B	109.6
C5—C6—H6A	109.7	H18A—C18A—H18B	108.1
C5—C6—H6B	109.7	C19A—C18A—C17	110.5 (4)
C5—C6—C7	109.88 (18)	C19A—C18A—H18A	109.6
H6A—C6—H6B	108.2	C19A—C18A—H18B	109.6
C7—C6—H6A	109.7	C18A—C19A—H19A	110.0
C7—C6—H6B	109.7	C18A—C19A—H19B	110.0
C6—C7—H7A	109.3	H19A—C19A—H19B	108.4
C6—C7—H7B	109.3	C20—C19A—C18A	108.6 (4)
H7A—C7—H7B	107.9	C20—C19A—H19A	110.0
C8—C7—C6	111.78 (18)	C20—C19A—H19B	110.0
C8—C7—H7A	109.3	C17—C18B—H18C	108.8
C8—C7—H7B	109.3	C17—C18B—H18D	108.8
C7—C8—H8A	109.2	H18C—C18B—H18D	107.7
C7—C8—H8B	109.2	C19B—C18B—C17	113.9 (5)
C7—C8—C9	112.01 (19)	C19B—C18B—H18C	108.8
H8A—C8—H8B	107.9	C19B—C18B—H18D	108.8
C9—C8—H8A	109.2	C18B—C19B—H19C	108.7
C9—C8—H8B	109.2	C18B—C19B—H19D	108.7
C8—C9—H9A	109.6	C18B—C19B—C20	114.3 (5)
C8—C9—H9B	109.6	H19C—C19B—H19D	107.6
H9A—C9—H9B	108.1	C20—C19B—H19C	108.7
C10—C9—C8	110.42 (17)	C20—C19B—H19D	108.7
C10—C9—H9A	109.6	C19A—C20—H20A	109.5
C10—C9—H9B	109.6	C19A—C20—H20B	109.5
C5—C10—C9	122.02 (18)	C19B—C20—H20C	110.2
C11—C10—C5	106.60 (18)	C19B—C20—H20D	110.2
C11—C10—C9	131.39 (19)	H20A—C20—H20B	108.1
C10—C11—H11	125.9	H20C—C20—H20D	108.5
C10—C11—C12	108.14 (18)	C21—C20—C19A	110.5 (3)
C12—C11—H11	125.9	C21—C20—C19B	107.6 (3)
C13—C12—C11	131.04 (19)	C21—C20—H20A	109.5
C13—C12—N4	121.26 (18)	C21—C20—H20B	109.5
N4—C12—C11	107.70 (17)	C21—C20—H20C	110.2
C12—C13—C23	118.80 (19)	C21—C20—H20D	110.2
C14—C13—C12	120.44 (19)	C16—C21—C20	125.0 (2)
C14—C13—C23	120.71 (18)	N22—C21—C16	110.09 (18)
C13—C14—C15	131.68 (19)	N22—C21—C20	124.87 (19)
C13—C14—N22	120.88 (18)	C13—C23—H23A	109.5
N22—C14—C15	107.43 (18)	C13—C23—H23B	109.5
C14—C15—H15	125.9	C13—C23—H23C	109.5
C16—C15—C14	108.12 (19)	H23A—C23—H23B	109.5
C16—C15—H15	125.9	H23A—C23—H23C	109.5
C15—C16—C17	131.3 (2)	H23B—C23—H23C	109.5
C15—C16—C21	106.56 (19)	N4—B1—N22	106.61 (16)
C21—C16—C17	122.11 (19)	F2—B1—N4	110.69 (18)
C16—C17—H17A	109.8	F2—B1—N22	109.88 (18)
C16—C17—H17B	109.8	F2—B1—F3	109.34 (17)
C16—C17—H17C	109.3	F3—B1—N4	110.28 (18)
C16—C17—H17D	109.3	F3—B1—N22	110.01 (18)
C16—C17—C18A	109.4 (3)	C5—N4—C12	107.41 (17)
H17A—C17—H17B	108.2	C5—N4—B1	127.50 (17)
H17C—C17—H17D	108.0	C12—N4—B1	125.08 (17)
C18A—C17—H17A	109.8	C14—N22—B1	125.64 (17)
C18A—C17—H17B	109.8	C21—N22—C14	107.80 (17)
C18B—C17—C16	111.6 (3)	C21—N22—B1	126.57 (17)
C18B—C17—H17C	109.3		
			
C5—C6—C7—C8	43.9 (2)	C17—C16—C21—C20	1.6 (3)
C5—C10—C11—C12	−0.4 (2)	C17—C16—C21—N22	−179.13 (19)
C6—C5—C10—C9	0.7 (3)	C17—C18A—C19A—C20	67.8 (6)
C6—C5—C10—C11	−179.60 (19)	C17—C18B—C19B—C20	−60.8 (8)
C6—C5—N4—C12	−179.95 (18)	C18A—C17—C18B—C19B	−49.4 (9)
C6—C5—N4—B1	1.1 (3)	C18A—C19A—C20—C19B	41.9 (6)
C6—C7—C8—C9	−63.7 (3)	C18A—C19A—C20—C21	−48.3 (5)
C7—C8—C9—C10	47.5 (3)	C19A—C20—C21—C16	15.5 (4)
C8—C9—C10—C5	−17.1 (3)	C19A—C20—C21—N22	−163.7 (3)
C8—C9—C10—C11	163.3 (2)	C18B—C17—C18A—C19A	50.6 (10)
C9—C10—C11—C12	179.2 (2)	C18B—C19B—C20—C19A	−55.1 (7)
C10—C5—C6—C7	−14.0 (3)	C18B—C19B—C20—C21	45.7 (6)
C10—C5—N4—C12	0.5 (2)	C19B—C20—C21—C16	−17.3 (4)
C10—C5—N4—B1	−178.41 (19)	C19B—C20—C21—N22	163.5 (3)
C10—C11—C12—C13	−178.9 (2)	C20—C21—N22—C14	178.4 (2)
C10—C11—C12—N4	0.7 (2)	C20—C21—N22—B1	−1.6 (3)
C11—C12—C13—C14	179.6 (2)	C21—C16—C17—C18A	15.2 (4)
C11—C12—C13—C23	2.3 (3)	C21—C16—C17—C18B	−12.2 (4)
C11—C12—N4—C5	−0.8 (2)	C23—C13—C14—C15	−1.1 (3)
C11—C12—N4—B1	178.19 (19)	C23—C13—C14—N22	177.37 (19)
C12—C13—C14—C15	−178.4 (2)	N4—C5—C6—C7	166.52 (19)
C12—C13—C14—N22	0.1 (3)	N4—C5—C10—C9	−179.72 (18)
C13—C12—N4—C5	178.88 (18)	N4—C5—C10—C11	−0.1 (2)
C13—C12—N4—B1	−2.2 (3)	N4—C12—C13—C14	0.1 (3)
C13—C14—C15—C16	178.4 (2)	N4—C12—C13—C23	−177.26 (18)
C13—C14—N22—C21	−178.18 (18)	N4—B1—N22—C14	−3.3 (3)
C13—C14—N22—B1	1.8 (3)	N4—B1—N22—C21	176.75 (17)
C14—C15—C16—C17	179.5 (2)	N22—C14—C15—C16	−0.2 (2)
C14—C15—C16—C21	−0.3 (2)	N22—B1—N4—C5	−177.86 (18)
C15—C14—N22—C21	0.6 (2)	N22—B1—N4—C12	3.4 (3)
C15—C14—N22—B1	−179.37 (18)	F2—B1—N4—C5	−58.4 (3)
C15—C16—C17—C18A	−164.6 (3)	F2—B1—N4—C12	122.9 (2)
C15—C16—C17—C18B	168.0 (3)	F2—B1—N22—C14	−123.3 (2)
C15—C16—C21—C20	−178.5 (2)	F2—B1—N22—C21	56.8 (3)
C15—C16—C21—N22	0.7 (2)	F3—B1—N4—C5	62.7 (3)
C16—C17—C18A—C19A	−49.1 (5)	F3—B1—N4—C12	−116.0 (2)
C16—C17—C18B—C19B	40.7 (7)	F3—B1—N22—C14	116.3 (2)
C16—C21—N22—C14	−0.8 (2)	F3—B1—N22—C21	−63.7 (3)
C16—C21—N22—B1	179.16 (19)		

Symmetry codes: (i) *x*+1, *y*, *z*; (ii) −*x*, −*y*+1, −*z*+1; (iii) −*x*+1, −*y*+1, −*z*+1.

### Hydrogen-bond geometry (Å, º)

*Cg*1 and *Cg*2 are the centroids of the N4,C5,C10–C12 and N22,C21,C14–C16 rings, respectively.

**Table:**

*D*—H···*A*	*D*—H	H···*A*	*D*···*A*	*D*—H···*A*
C23—H23*B*···F3^iv^	0.96	2.66	3.621 (3)	178
C8—H8*B*···F2^v^	0.97	2.56	3.252 (3)	129
C17—H17*A*···*Cg*2^iii^	0.97	3.10	3.879 (3)	138

Symmetry codes: (iii) −*x*+1, −*y*+1, −*z*+1; (iv) −*x*, −*y*+1, −*z*; (v) *x*−1/2, −*y*+1/2, *z*−1/2.
